# Experimental and bioinformatic approach to identifying antigenic epitopes in human α- and β-enolases

**DOI:** 10.1016/j.bbrep.2018.05.008

**Published:** 2018-06-17

**Authors:** Jadwiga Pietkiewicz, Regina Danielewicz, Iwona S. Bednarz-Misa, Ireneusz Ceremuga, Jerzy Wiśniewski, Magdalena Mierzchala-Pasierb, Agnieszka Bronowicka-Szydełko, Edmund Ziomek, Andrzej Gamian

**Affiliations:** a Department of Medical Biochemistry, Wroclaw Medical University,Chalubinskiego 10, 50-368 Wroclaw, Poland; b Wroclaw Research Center, Stablowicka 147, 50-066 Wroclaw, Poland

**Keywords:** AP, alkaline phosphatase, BSA, bovine serum albumin, ELISA, enzyme-linked immunosorbent assay, ESI, electrospray injection, HRP, horse radish peroxidase, IgG, immunoglobulin G, LC, liquid chromatography, MeOH, methanol, MS, mass spectrometry, OPD, ortho-phenylenediamine, PAGE, polyacrylamide gel electrophoresis, PBS, phosphate buffered saline, PMSF, phenylmethylsulfonyl fluoride, pNPP, para-nitrophenyl phosphate, SDS, sodium dodecylsulfate, TBST, 20 mM Tris, pH 7.4, 150 mM NaCl, 0.05% Tween-20, UPLC-Q-TOF-MS, ultrapressure liquid chromatography, quadrupole-time-of-flight mass spectrometer, WB, western blotting, Enolase purification, Mass spectrometry, Epitope prediction, Specific antibodies, Cross-reactivity

## Abstract

Human α- and β-enolases are highly homologous enzymes, difficult to differentiate immunologically. In this work, we describe production, purification and properties of anti-α- and anti-β-enolase polyclonal antibodies. To raise antibodies, rabbits were injected with enolase isoenzymes that were purified from human kidney (α-enolase) and skeletal muscle (β-enolase). Selective anti-α- and anti-β-enolase antibodies were obtained by affinity chromatography on either α- or β-enolase-Sepharose columns. On Western blots, antibodies directed against human β-enolase, did not react with human α-isoenzyme, but recognized pig and rat β-enolase. To determine what makes these antibodies selective bioinformatic tools were used to predict conformational epitopes for both enolase isoenzymes. Three predicted epitopes were mapped to the same regions in both α- and β-enolase. Peptides corresponding to predicted epitopes were synthesized and tested against purified antibodies. One of the pin-attached peptides representing α-enolase epitope (the C-terminal portion of the epitope 3 - S^262^PDDPSRYISPDQ^273^) reacted with anti-α-enolase, while the other also derived from the α-enolase sequence (epitope 2 - N^193^VIKEKYGKDATN^205^) was recognized by anti-β-enolase antibodies. Interestingly, neither anti-α- nor anti-β-antibody reacted with a peptide corresponding to the epitope 2 in β-enolase (G^194^VIKAKYGKDATN^206^). Further analysis showed that substitution of E^197^ with A in α-enolase epitope 2 peptide lead to 70% loss of immunological activity, while replacement of A^198^ with E in peptide representing β-enolase epitope 2, caused 67% increase in immunological activity. Our results suggest that E^197^ is essential for preserving immunologically active conformation in epitope 2 peptidic homolog, while it is not crucial for this epitope's antigenic activity in native β-enolase.

## Highlights

• The α- and β-enolase preparations were isolated from human tissues.
• Specific rabbit affinity-purified antibodies were obtained using immobilized human α- and β-enolases.
• Three conformational epitopes were predicted for both human enolases with aid of bioinformatic tools.
• Two peptides corresponding to predicted epitopes reacted with anti- enolase antibodies.

## 1 Introduction

Enolase (E.C. 4.2.1.11) is a dual function enzyme essential for cellular processes. It acts as 2-phospho-D- glycerate hydro-lyase in glycolysis pathway, and as phosphoenolpyruvate hydratase in gluconeogenesis pathway [1]. This highly conserved protein retains similar catalytic function in prokaryotes and eukaryotes [2]. In many organisms, including primates and lower mammals, enolase is responsible for both catabolic and anabolic processes. Enolase is enzymatically active as a dimer. Three types of subunits, α, β and γ, each encoded by a separate gene can form a dimer. Both homo- and heterodimers are formed. Expression of α, β and γ subunits is regulated developmentally and in a tissue-specific manner [1], [2], [3]. The α homodimer is found in human fetus and adult tissues such as lung, liver, adipose tissue, pancreas, spleen and kidney. The β isoenzyme is present in tissues with high energy requirements such as heart and skeletal muscle (αβ and ββ isoenzymes), while γ-enolase is found in neuronal and neuroendocrine cells (αγ and γγ isoforms). The ββ enolase accounts for 3% of soluble proteins in human skeletal muscle and more than 90% of overall enolase activity [3], [4].

Some highly conserved proteins sometimes perform multiple functions, often very different from their well-known “classical” activities. Such proteins were recently named “moonlighting” proteins [5]. A considerable number of glycolytic pathway enzymes, including enolase, exhibit non-glycolytic functions [6]. In eukaryotic cells enolase is primarily located in the cytosol, where besides its catalytic function it participates in a regulation of the cell morphology and is interacting with the cytoskeleton [7]. The enolase has also been detected in mammalian cell nuclei where it is participating in the transcriptional regulation of genes involved in cells’ morphological transformation and proliferation [8], [9]. The α-enolase has been implicated in numerous diseases [2], [10] including metastatic cancer [11], [12], neurodegenerative diseases [13], autoimmune disorders [14], [15], [16] and in bacterial infections [17]. Additionally, there are reports describing α-enolase as being expressed at the cell surface of nonpathogenic and pathogenic microorganisms [18], [19], [20]. The disease-related role of α-enolase is mostly attributed to its immunogenic capacity, DNA-binding ability, and plasmin/plasminogen receptor function [10]. It is important to note that enolase primary and tertiary structures are highly conserved. Alignment of human enolase isoenzymes shows approximately 83–84% of sequence identity and 91–93% of similarity. However, some structural differences between enolases of human tissue-specific variants and lower-mammals exist [2], [3], [21].

For all enolase isoforms, the active site and subunit interface is most conserved and composed of the same amino acids [2]. However, short variable region localized at the surface of the molecule is probably responsible for enolase interaction with other macromolecules [22]. Moreover, this variable region may also act as a site of the enolase distinct antigenic epitopes. This notion has been supported by the identification of non-cross-reacting antibodies recognizing different enolase isoenzymes in patients with auto-immunological diseases. The sera of patients with lupus erythematosus and hypophysitis do not react with rabbit muscle enolase but recognize human α-enolase [23], [24]. It is noteworthy to add that α-enolase is a common autoantigen in several autoimmune diseases [25], [26], [27], a feature related to noncatalytic properties of this protein. The α-enolase also functions as a plasminogen receptor, a modulator of the cell growth and differentiation by down-regulating c-myc protooncogene expression, a structural protein in the lens of some species, and possibly as a suppressive lymphokine [10], [15], [28], [29]. The mass spectrometry offers the best accuracy in detecting α-enolase in biological samples and in discriminating it from other proteins [30], [31]. However, the abundance of α-enolase in autoimmune diseases renders it less attractive as a biomarker.

On the other hand, the human muscle-specific enolase may become useful marker in diagnosis of the muscule-related diseases. During cardiac and skeletal muscle development the β-enolase progressively replaces α-isoform and becomes an early marker of the myogenic differentiation [7], [32]. For this reason, β-enolase was described as a sensitive histological marker in rhabdomyosarcoma [4]. Muscle-specific enolase deficiency was observed in metabolic myopathy [33]. An increase of the β isoenzyme occurs in the serum of the patients with both acute myocardial infarction and exercise-induced muscle damage [34], [35]. Furthermore, detection of the β-enolase in bloodstains after traumatic skeletal muscle injury has a potential to be used in a forensic practice [36]. Only a few reports refer to immunochemical detection of β-enolase in biological material [32], [35], [36], [37], [38]

In this work bioinformatic and empirical approaches were used to identify antigenic epitopes in human α- and β-enolases. The gained knowledge was used to raise specific antibodies against these two enolases. Polyclonal antibodies recognized some of the predicted epitopes.

The gained knowledge will aid us in obtaining specific α-and β-enolase monoclonal antibodies and help in developing diagnostic test enabling differentiation of these two isoenzymes.

## 2 Materials and methods

### 2.1 General

All chemicals were of the analytical grade. 2-Phospho-D-glycerate was purchased from Fluka (FlukaAnalytical, St. Gallen, Switzerland). The molecular mass protein markers for SDS-PAGE were from Bio-Rad (Bio-Rad Polska, Warsaw, Poland). Other reagents were purchased from Sigma-Aldrich (Sigma-Aldrich Poland, Poznan, Poland). LC/MS-grade acetonitrile, water, and formic acid were from Baker Company (J.T. Baker, Griesheim, Germany). Secondary antibodies for ELISA and Western blotting were purchased from Promega (Promega Corp., Madison, USA). The Horse Radish Peroxidase (HRP) substrate, o-phenylenediamine, was acquired from Promega, while the alkaline phosphatase (AP) substrate, p-nitrophenyl phosphate, came from Sigma-Aldrich. The EZ-Link™ NHS-PEG Solid Phase Biotynylation Kit: *spin columns* and fluorescein-labeled streptavidin were obtained from Thermo Scientific (Thermo Scientific, Rockford, USA).

The human tissues for enolase purification were obtained as histologically normal tissues from Department of General, Vascular and Transplantation Surgery of Wroclaw Medical University. Kidney as a blood-group mismatched and not immunogenic organ from a healthy individual (positive opinion No. KB-516/2005 issued by the Bioethics Committee of Wroclaw Medical University) and samples of human tibialis anterior muscle as postoperative material, in accordance with Polish legal requirements under the license issued by the Commission of Bioethics of Wroclaw Medical University (positive opinion No. KB-516/2005). The fresh rat skeletal muscle samples were obtained from the Animal Laboratory of the Wroclaw Medical University, and pig skeletal muscle samples were purchased at the local slaughterhouse. New Zealand rabbits were purchased from local rabbit breeding farm. All experiments involving rabbits were performed at the animal facility at the Institute of Immunology and Experimental Therapy, Polish Academy of Sciences in Wroclaw. Animals were cared for in accordance with criteria approved by the Institutional Animal Ethical Committee of the Institute of Immunology and Experimental Therapy, Polish Academy of Sciences in Wroclaw (LKE 53/2009).

### 2.2 Purification of α- and β-enolases

At all stages of the enzyme purification, the magnesium sulfate and temperature set at 4 °C were used to prevent the loss of the enolase activity. Magnesium ions are essential for both catalysis and stability of the enolase dimer [2], [39]. Purification of β-enolase from human muscle tissue was performed according to the procedure described earlier [40]. Briefly, 110 g of frozen striated muscle was homogenized in deionized water containing 3 mM MgSO_4_ and protease inhibitors PMSF (phenylmethylsulfonyl fluoride) and aprotinin (2 µg/ml). Homogenization was performed in PT3100D homogenizer (DanLab, Poland) at 4 °C for 10 min. The crude protein extract was incubated at 53–54 °C for 3 min, then cooled to 4 °C and denatured proteins removed by centrifugation at 9000 g for 45 min. The supernatant was fractionated with ammonium sulfate at 4 °C, by adding solid salt in small portions and by stirring gently. The mixture was left overnight at 4 °C after reaching 60% saturation of (NH_4_)_2_SO_4_ and precipitated proteins were centrifuged at 9000 g for 45 min and discarded. Protein precipitation was continued by adding solid (NH_4_)_2_SO_4_ up to 80% saturation, and after overnight storage at 4 C the precipitated proteins were centrifuged. The pellet was dissolved in 20 mM Tris-HCl buffer pH 9.0, containing 3 mM MgSO_4_ and 1 mM β − mercaptoethanol and dialyzed against this buffer. In the following step, the sample was loaded on a DEAE-Sephadex A-50 column (30 × 3 cm) equilibrated with the same buffer. Under these conditions, β-enolase did not adhere to this ion-exchanger, and fractions containing enolase activity were eluted (0.08 ml/min flow-rate) with equilibrating buffer and precipitated with ammonium sulfate. The pellet was dissolved in 10 mM phosphate (Na+) buffer pH 6.4, containing 3 mM MgSO_4_, and it was applied to a CM-Sephadex C-50 column (10 × 3 cm) equilibrated with the same buffer. After elution of the bound proteins with a pH gradient of 6.4–9.0 (0.08 ml/min flow-rate), fractions with enolase activity were pooled, concentrated and next fractionated on a QAE-Sephadex column (5 × 1.5 cm) in 20 mM Tris-HCl buffer pH 9.0, containing 3 mM MgSO4 and 1 mM β-mercaptoethanol. The main peak, with enolase activity, was collected and precipitated by dialysis in 80% ammonium sulfate. The pellet dissolved in 7.5 mM imidazole-HCl buffer pH 6.8, containing 2.5 mM MgSO_4_, 50 mM NaCl and 50% glycerol, was stored at 4 °C for several months without loss of activity. For comparative immunological experiments, pure or partially purified (i.e., after DEAE-Sephadex fractionation) β-enolase from pig and rat muscles were used.

The above described β-enolases purification protocol procedure was adapted for isolation of α-enolase from human kidney. The 62 g of kidney was homogenized for 10 min in 3 volumes of 20 mM Tris-HCl buffer pH 7.2 containing 3 mM MgSO_4_, 2 mM β-mercaptoethanol, with PMSF (4 µg/ml) and aprotinin (2 μl/10 ml) as protease inhibitors (buffer A). The crude extract was centrifuged at 4500 g for 45 min at 4 °C and filtered for fats removal. The thermal denaturation step was omitted. Also, desalting of α-enolase was achieved at a lower concentration of (NH_4_)_2_SO_4_ (45–67%) than that for the muscle-specific enzyme. The precipitated protein was centrifuged at 20,000 g for 45 min at 4 °C, and the precipitate dissolved in 20 mM Tris-HCl buffer pH 9.0 containing 3 mM MgSO_4_ and 2 mM β-mercaptoethanol (buffer B). After dialysis at 4 °C against the buffer B (thrice for 4 h), the sample was loaded on DEAE-Sephadex A-50 column (30 × 3 cm) equilibrated with the same buffer. The column was washed with buffer B (0.08 ml/min flow rate), and fractions containing enolase were pooled and concentrated using Amicon Ultra 15, 30 kDa membranes. Next, the protein solution was dialyzed against 20 mM phosphate buffer pH 6.0 containing 3 mM MgSO_4_ with 1 mM β-mercaptoethanol (buffer C). After dialysis the sample was subjected to column chromatography on CM-Sephadex C-50 (10 × 3 cm) equilibrated with buffer C. The bound enolase was eluted (0.08 ml/min) with pH gradient 6.0–8.0 in buffer C. The fractions with active enzyme were collected and concentrated on Amicon Ultra 15, 30 kDa Falcon concentrators. After dialysis against the stabilizing buffer (20 mM imidazole buffer, pH 7.0 with 3 mM MgSO_4_ and 10% glycerol) the human α-enolase was stored at − 20 °C. All column chromatography steps were performed on the FPLC ӒKTAexplorer system (Amersham, Pharmacia Biotech, Upsala, Sweden). The purification summary of α- and β-enolase is listed in Supplementary Table 1. Purity of the enolase samples at different stages of purification is shown on Supplementary Fig. 1.

### 2.3 Enolase purity assessment by SDS-PAGE

The SDS-PAGE of the enolase samples was carried out on 10% gels using Bio-Rad system [41]. Proteins were stained with 0.25% Coomassie Brilliant Blue (CBB) R250 in 10% acetic acid with 40% methanol and the gels were destained in 5% methanol in 7.5% acetic acid. The VilberLourmat System and BIO 1D+ software were used to obtain and process the gel images.

### 2.4 Enolase purity assessment by mass spectrometry

The mass spectrometric studies were conducted using NanoAcquityUPLCQ-TOF/MS (Waters Corp., Milford, MA, USA) system. Nano Ultra Performance Liquid Chromatograph was equipped with a BEH 300 C4 analytical column (100 mm × 100 µm; 1.7 µm, USA) and Symmetry C18 precolumn (180 µm × 20 mm; 1.7 µm, USA) equilibrated with 15% acetonitrile (solvent B) in water (solvent A). Both solvents A and B contained 0.1% TFA. The enolase was adsorbed on the precolumn, and the column was washed with 15% solvent B for 3 min with the flow-rate maintained at 400 nL/min. The analytical column temperature was set to 35 °C, and it was washed at the 15% B per min. gradient15–96% B for 15 min, followed by 5 min wash with 96% B and it returned to wash with 15% B for the next 10 min. Eluted and desalted enolase was analyzed byXevo G2-Q-TOF (Waters Corp., Milford, MA, USA) mass spectrometer equipped with a nano-electrospray ionization source. The capillary voltage was set at 3.0 kV, and the cone voltage for enolase at 40 V. The cone gas flow was maintained at 40 L/h, and the source temperature was set at 80 °C. The nanoflow gas pressure was set at 0.18 bar. The system was set at the positive ion sweep. The [Glu1]-Fibrinopeptide B human (Waters Corp., Milford, MA, USA) was used as the lock mass solution in the accurate mass measurement mode. Data were collected from *m/z* 200 to *m/z* 2000. Deconvolution of mass spectra was performed with MaxEnt 1 (Waters Corp., Milford, MA, USA).

### 2.5 Rabbit anti-human enolase antibodies

Immunization of rabbits with either human α-, or β-enolase preparations and isolation of the antibodies was done according to earlier described protocol [42]. Briefly, the first subcutaneous multipoint injection was performed with the mixture of 0.25 ml of 0.6% antigen in of PBS with 0.25 ml of complete Freund's adjuvant. The mixture (0.5 ml) of 0.6% antigen in PBS mixed in 1:1, (v: v) ratio with incomplete Freund's adjuvant was injected twice in two weeks intervals. Ten days after the last injection animals were bled and serum incubated at 56 °C for 30 min to inactivate complement. Immunoglobulins were precipitated twice with 50% saturated ammonium sulfate [43]. For the anti-α-enolase antibodies, ammonium sulfate concentration was lowered to 33–40% saturation. The pellet was dissolved in 10 mM phosphate buffer (K^+^), pH 6.8 and after dialysis proteins were further fractionated on a DEAE-Cellulose equilibrated with the same buffer. Fractions were assayed by double immunodiffusion in 1% agarose [44], and active fractions were pooled for further purification. Finally, anti-α- and β-enolase antibodies were purified by immunoaffinity chromatography using respective antigens immobilized to Sepharose-4B [42]. Antibodies were eluted from the column (5 × 1.6 cm) with 3 M KCNS with flow rate 0.08 ml/min, dialyzed against PBS, concentrated by ultrafiltration and stored at − 20 °C in 50% glycerol.

### 2.6 Prediction of the antigenic determinants in α- and β-enolases

The B-cell type antigenic epitopes were predicted using method relying on α- and β-enolases’ 3D structural features [45] – see also http://pepito.proteomics.ics.uci.edu/. The enolase structures of α- and β-enolase (PDB ID: 2PSN and 2XSX, respectively) were used for the analysis. The regions with the highest score and with amino acid side-chains exposed to protein's surface were selected.

### 2.7 Synthesis of peptides tethered to polyethylene pins

The peptides corresponding to predicted epitopes were synthesized using NCP Block of 96 hydroxypropylmethacrylate pins (MIMOTOPES, Clayton, Victoria, Australia) according to earlier described protocol [46]. The synthesis was performed in 96-well plates, following the one-pin – one-peptide approach. Each pin was submerged in 100 μl of the solution containing 60 mM F-moc amino acid and an equimolar amount of diisopropylcarbodiimide and N-hydroxybenzotriazole as coupling reagents. Each coupling cycle lasted 4 h., and to assess the completion of the coupling reaction; pins were tested with 10 mM bromophenol blue for the presence of free amino groups. Peptides were deprotected and pins washed with MeOH, 0.5% acetic acid in MeOH, MeOH, dried, and stored at − 20 °C. The pins were treated with “disruption buffer” composed of 1% SDS, 0.1% 2-mercaptoethanol and 0.1 M Na_3_PO_4_, pH 7.2 to prepare them for the enzyme-linked immunosorbent assay (ELISA). For that purpose pins were heated to 60 °C and placed in a sonication bath for 10 min. To remove the disruption buffer, pins were briefly rinsed with water and placed in a 60 °C water-bath for 30 min. Before ELISA pins were equilibrated with Tween 20 in Tris-buffered saline (TBST) for 10 min at room temperature.

### 2.8 Determining antibody titer

ELISA was performed on polystyrene plates (MaxiSorp, Nunc, Roskilde, Denmark). The wells were coated with 0.5 µg of human α- or β-enolase dissolved in 100 μl of 50 mM carbonate-bicarbonate buffer, pH 9.6 [47]. The plates were incubated at 37 °C for 4 h. and at 4 °C overnight. Wells were washed three times with TBST (20 mMTris,150 mM NaCl, pH 7.4 containing 0.05% Tween-20), to remove the excess of the antigen. Unoccupied protein binding sites on the polystyrene plate were blocked with 3% fat-free skimmed milk in TBS (20 mM Tris, 150 mM NaCl, pH 7.4) at 37 °C for 1 h. After washing with TBST, antibodies against human α-enolase or β-enolase were added in series of dilutions ranging from 0.003 μg/ml to 25 μg/ml and incubated at 37 °C for 2 h. The primary antibody was removed and after washing with TBST, the secondary antibody, goat anti-rabbit IgG-Horse Radish Peroxidase (HRP) conjugate (Promega) at 1:3000 dilution was added and incubated for 2 h. at 37 °C. After an exhaustive washing with TBST, peroxidase activity was determined using o-phenylenediamine (OPD) as the substrate. Absorbance was monitored at 450 nm in a PerkinElmer microplate reader for duplicate samples. Additionally, the ELISA was used to assess cross-reactivity of α-enolase with anti- β-enolase antibodies, and β-enolase with anti-α -enolase antibodies. In this experiment, antibodies against α-enolase were diluted at1:3000 and those against β-enolase at 1:300.

### 2.9 ELISA test with pin-tethered peptides

The TBST soaked pins were transferred to a 96-well plate filled with 200 μl of 1% BSA in TBST and incubated for 60 min at room temperature [46]. After blocking step, pins were immersed in 100 μl of anti-enolase antibody solution and incubated at room temperature for 60 min. The antibody preparation was diluted with 1% BSA in TBST. The antibody against β-enolase was diluted 100-fold, while anti-α-enolase antibodies 1000-fold to assure comparable titers for both working samples. Next, pins were washed three times, each time for 5 min in 10 ml of TBST. The attached antibodies were detected after incubation of the pins with goat-anti-human IgG-alkaline phosphatase (AP) conjugate (ICN Biomedicals, Aurora, Ohio, USA, diluted according to the manufacturer's recommendations). The AP activity was measured to determine the level of the bound conjugate. The p-nitrophenyl phosphate (pNPP Liquid substrate for ELISA, Sigma) was used as the AP substrate. The enzymatic reaction was stopped by removal of pins from the plate, and the amount of released p-nitrophenol was measured at 406 nm in a plate reader (PerkinElmer). To re-use pins, they were treated with the “disruption buffer” as described below.

### 2.10 Immunoblotting

The proteins were transferred from polyacrylamide gel onto an Immobilon P membrane (transblot system Bio-Rad, Poland), under conditions described elsewhere [42]. The membrane with transferred proteins was incubated at 37 °C for 1 h with affinity-purified biotinylated antibodies against either human α- or β-enolase. Biotinylation of the antibodies (0.5 mg IgG) was performed using The EZ-Link™ NHS-PEG Solid Phase Biotinylation Kit: *spin columns* (Thermo Scientific, Rockford, USA) according to producer instruction available at www.thermoscientific.com./pierce. Biotinylated product was stored at 4 °C in 0.2 M imidazole in PBS. For the purpose of immunobloting both biotinylated-antibody samples were diluted with TBST to the equal titer (1:3000 and 1:300 for anti-α-enolase antibody and anti-β-enolase antibody, respectively). After the protein electrotransfer, the Immobilon P membrane was blocked overnight with 1% Tween-20, washed with TBST 01% and incubated at room temperature for 1 h with biotinylated antibodies against human α-enolase or human β-enolase. The excess of antibodies was removed by washing the membrane with TBST (4 × 10 min), followed by 45 min incubation of the membrane with streptavidin-fluorescein conjugate (1:5000 dilution) in the dark. After washing off unbound conjugate with water, the detection of proteins fluorescence was performed in GBox EF2 multi-application imaging system (SYNGENE, UK) with GeneSys version 1.3.10 software.

To perform the dot-blot, Immobilon P membrane was activated for 15 s in 100% methanol, soaked in distilled water for 2 min and followed directly by 5 min equilibration in TBST. A sample of 2 μl of each protein was spotted within a pre-marked grid and dried for 2 h. The membrane was blocked for 30 min with 2% casein in TBST and incubated 30 min with primary antibody, i.e., anti-α-enolase or anti-β-enolase antibodies diluted 1:5000 and 1:500, respectively. The blots were treated with the goat anti-rabbit IgG conjugated with alkaline phosphatase secondary antibody (1:5000) and the blot was developed by incubating membrane with the alkaline phosphatase substrate Western BlueR (Promega), and the reaction was stopped with water.

### 2.11 Conjugation of synthetic peptides with myoglobin

Synthetic peptides, corresponding to predicted antigenic epitopes were coupled to horse myoglobin. Before conjugation, all accessible free amino groups on the protein's surface were blocked with the bromoacetic acid N-hydroxysuccinimide ester. The degree of the amine group derivatization was monitored with 2,4,6-trinitrobenzenesulfonic acid (Fluka, Buchs, Switzerland). The SH-groups in cysteinylated peptides were protected with trimethylphosphine (Sigma-Aldrich, 1 M solution in THF) to prevent the formation of disulfide bonds. The reaction conditions for peptide-myoglobin conjugation via thioether bonds were adopted from a report describing the chemical coupling of polysaccharides to this carrier protein [48].

### 2.12 Surface plasmon resonance (SPR)

The binding studies of the antibodies against human α- or β- enolase with their corresponding native antigens or selected synthetic bioconjugates were performing using BIAcore T200 system (GE Healthcare AB, Uppsala, Sweden). All experiments were carried out on Sensor Chip Protein A (GE Healthcare, Uppsala, Sweden) at room temperature. Always 10 mM HEPES pH 7.0 containing 150 mM NaCl, 3 mM EDTA and 0.005% detergent P20 was used as a running buffer and sample dilution buffer. Ligands, i.e., antibodies against human α-enolase or human β-enolase were immobilized on sensor chip at a flow rate 5 μl/min. Dilution of antibody against β-enolase and anti α-enolase was similar to that used in ELISA assay with pin-tethered peptides. Before performing binding studies, the antibodies immobilized Protein A sensor surface was equilibrated with running buffer. The natural antigens, i.e., α-enolase or β-enolase and two conjugates of myoglobin with synthetic peptides, i.e., epitope 2 for α-enolase ([N^193^VIKEKYGKDATN^205^]) and C-terminal part of the α-enolase epitope 3 (S^262^PDDPSRYISPDQ^273^) were used at 26 nM concentration. Each analyte was injected for 6 min at a flow rate of 30 μl/min. The association phase was run for 120 s and the dissociation of the bound analyte from Ab was studied for 500 s. At the end of each cycle, the antibody surface was regenerated by injecting 10 mM glycine-HCl pH 1.5. BIA evaluation software version 3 was used for binding kinetics determination according to 1:1 Langmuir isotherm.

### 2.13 Determination of protein concentration

The concentration of purified enolase was determined spectrophotometrically at 280 nm using absorption coefficient A 0.1% = 0.89 established for rabbit muscle enolase [1]. The Lowry method was applied to determine the concentration of the antibodies [49].

### 2.14 Statistics and data analysis

The Student's *t*-test was used to assess the statistical significance of the differences between the mean values of three or four independent experiments by using Statistica 12 software. All cases, where the value of p < 0.05 was determined, were accepted as significant.

## 3 Results and discussion

### 3.1 Purification of human α- and β-enolases

The ion-exchange chromatography was used to purify both α- and β-enolases. The method employed in this work takes advantage of different isoelectric point values calculated for human α- and β-enolases (7.38 and 7.72, respectively), muscle-specific enzymes from rat (7.45) and pig (8.8–9.0) [3], [21]. The summary of human enolase isoenzymes purification is presented in Supplementary Table 1. The purity of the enolase preparations was examined at every purification step by SDS-PAGE and is shown on Supplementary Fig. S1.

The quality of purified α- and β-enolase preparations was further assessed by mass spectrometry. The monoisotopic molecular mass values were determined experimentally and compared to their corresponding theoretical values (Table 1). The ESI-MS spectra are shown in Supplementary Fig. S2. All four enolase preparations show some difference between theoretical and empirical monoisotopic molecular mass values. In three out of four preparations listed in Table 1, the empirical molecular mass value, for a predominant component, differ from the theoretical by 45–47 Da. The post-translational modifications were not identified experimentally. A fair assumption, however, can be made that both human enolases were oxidized, since no reducing agent was used throughout the purification. It should be noted that the absence of the reducing agent had no impact on enolase's enzymatic viability. It is much harder to explain what might have caused a decrease in the molecular mass for the pig β-enolase. Among probable causes could be amino acid truncation at either C- or N-termini, combined with protein oxidation. Identity of the enolases was verified by ESI-MS-MS analyses of the tryptic digests. Although, the mass spectra shown in Table 1 and Supplementary Fig. S2 do not imply cross-contamination of β-enolase preparation with α-enolase, the analysis of the trypsinized sample (not shown) revealed trace amounts of α-enolase.

**Table 1:** Molecular mass determination.

Enolase preparation	Experimental data [Da]	Theoretical values [Da]	Gene Bank Accession No
Human α-enolase	47 080.40±0.2	47 035.87	NC_000001
	47 153.00±0.5		
Human β-enolase	46 900.50± 0.2	46 853.69	AC_000149
	46 845.03± 0.3		
Pig β-enolase	47 026.80±0.1	46 980.93	NC_010454
Rat β-enolase	46 925.50±0.1	46 880.81	NC_005109

The enolase samples were tested on NanoAcquityUPLCQ-TOF/MS (Waters Corp., Milford, MA, USA) system, under conditions described in Materials and Methods.

### 3.2 Immunological activity of anti-enolase polyclonal antibodies

To raise polyclonal antibodies, rabbits were either immunized with purified human α- or, β-enolase preparation. The immunization protocols were outlined in the Materials and Methods. Immunoglobulins were precipitated from rabbit sera with ammonium sulfate, dialyzed and further purified by anion-exchange chromatography on DEAE-Sephadex. The most active fractions, as determined by double-immunodiffusion test (not shown), were selected for further purification by affinity chromatography on either Sepharose-(α-enolase) or Sepharose-(β-enolase) column. Affinity purified antibodies against α-enolase (0.35 mg/ml) and β-enolase (0.49 mg/ml) were obtained.

**Fig. 1:**
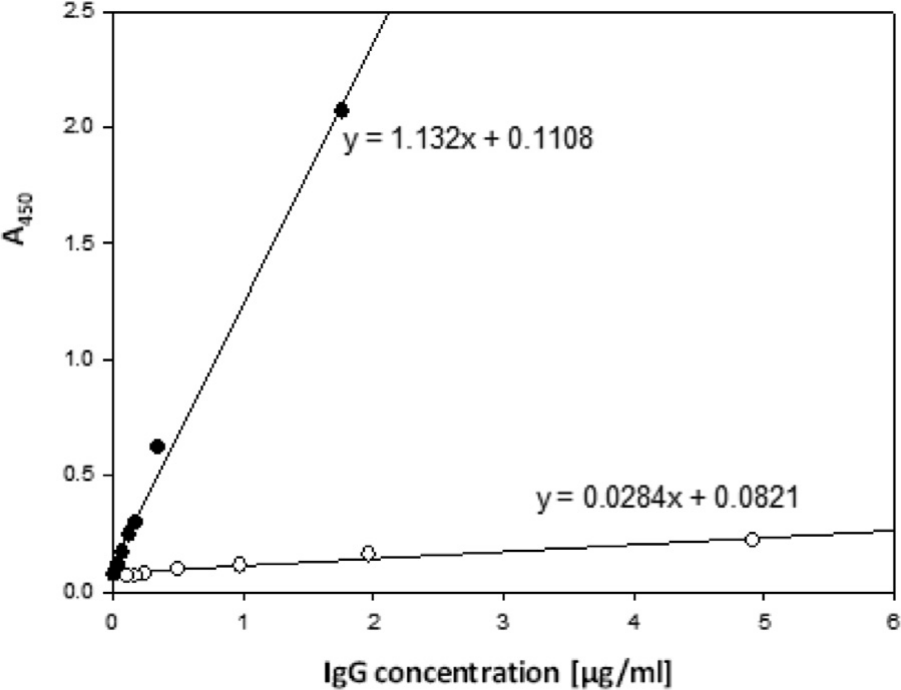
Immunological activity of the antibody preparations directed against human α-enolase (•―•) and β-enolase (о―о). ELISA plate wells were coated with 0.5 μg of purified α-, or β-enolase. The plates were washed, blocked with TBST and allowed to react with series of diluted, affinity chromatography purified, anti-human-α- and β-enolase antibody preparations. Goat anti-rabbit-IgG-HRP conjugate was used as the secondary antibody at 1:3000 dilution. Formation of the immunological complexes was tested by measuring HRP activity with *o*-phenylenediamine (OPD) as the HRP substrate. The progress of the HRP activity was monitored at 450 nm in a PerkinElmer microplate reader. Results were plotted and fitted into the linear regression equation. Slopes were calculated and compared. The data points are average of three experiments (n = 3) and average deviation from the mean for these data points was calculated at ± 0.015.

Despite similar protein concentration, these two preparations showed a quite dramatic difference in immunological activity (Fig. 1). Series of dilutions prepared for both IgG preparations were tested by ELISA. The data points are average of three experiments (n = 3) and average deviation from the mean for these data points was calculated at ± 0.015. It is also worth pointing out that the data points shown on Fig. 1 fit well into the linear regression equation and that the lines drawn through these points intercept at zero.

**Fig. 2:**
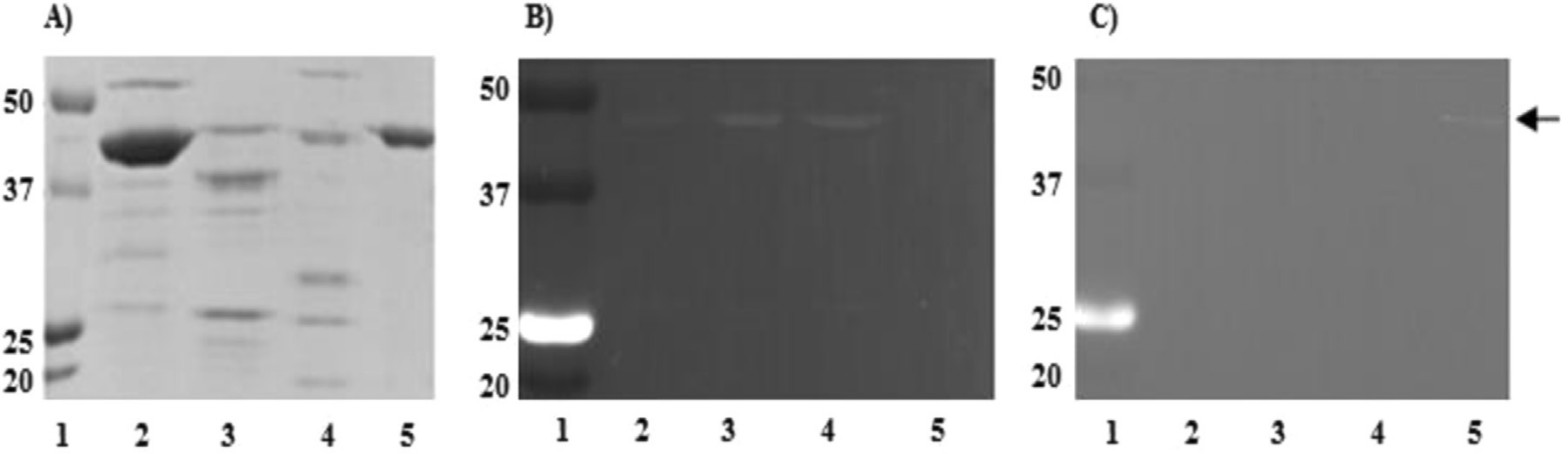
Cross-reactivity of anti-human-α- and β-enolase antibody preparations. (A) 10% SDS-PAGE gel, (B) Western blot of enolase detected with anti-human-β-enolase, (C) Western blot of enolase detected with anti-human-α-enolase. Both anti-human α- and β-enolase antibodies were biotinylated (see Materials and Methods) and were allowed to react with streptavidin-fluorescein conjugate (Thermo Scientific, Rockford, USA). The fluorescence was visualized in the GBox EF2 multi-application imaging system (SYNGENE, UK) and images were processed with GeneSys version 1.3.10 software. Lane 1 – MW markers; Lanes 2,3,4 – partially purified β-enolase preparations from a pig, rat, and human muscle, respectively; lane 5 – purified α-enolase from human kidney.

The results showed that anti-α-enolase antibody was 10-fold more active than the one directed against β-enolase. Such divergence in immunological activity can be primarily explained by the existence of two separate epitopes and/or a difference in binding constants. Recognition of the same epitope, but at a different affinity would have resulted in cross-reactivity between these two purified antibody preparations. To verify this assumption, both cross-reactivity, and specificity of anti-α- and anti-β-enolase antibodies were tested. In order to characterize the specificity of antibodies, the method based on detecting fluorescence signal of immunocomplexes was used. Both antibodies were labeled with biotin and allowed to react with streptavidin-fluorescein conjugate. This approach eliminates the need for the secondary antibody without sacrificing method's sensitivity. As shown in Fig. 2, no cross-reactivity can be seen between biotinylated anti-human α-enolase antibodies and β-enolase from a pig, rat, and human (Fig. 2C, lanes 2, 3 and 4, respectively). Only positive signal observed was the one for human α-enolase (Fig. 2C, lane 5). In addition, biotinylated antibodies against human-β-enolase could recognize pig, rat and human β-enolase (arrow at Fig. 2B, lanes 2, 3 and 4, respectively), but did not interact with α-enolase from human (Fig. 2B, lane 5). No interaction was observed between anti-β-enolase antibodies with other proteins extracted from striated muscle, as shown in Supplementary Fig. S3 for human and rat tissue.

The cross-reactivity was also tested by ELISA assay (Table 2). Affinity antibody preparations did not show cross-reactivity with native antigens under ELISA conditions.

**Table 2:** Cross-reactivity of human α- and β-enolase with affinity-purified rabbit anti-enolase antibodies.

Native antigen	Antibody	Antibody dilution		A_450_
α-enolase	Anti- α-enolase	1: 3000		0.183
β-enolase	Anti- α-enolase	1: 3000		0.020
α-enolase	Anti- β-enolase	1: 300		0.012
β-enolase	Anti- β-enolase	1: 300		0.142

Cross-reactivity of anti-α-enolase and anti-β-enolase affinity chromatography.

purified preparations was determined by ELISA (Materials and Methods).

while using goat anti-rabbit IgG conjugated with HRP (1: 3000).

When affinity of the antibodies was tested by SPR, the anti-α-enolase antibodies showed a very high affinity towards human α-enolase (K_D_ 5.55 ×10^–11^M) and much lower affinity towards β-enolase (K_D_ 3.44 × 10^−5^ M). The anti-β-enolase antibodies bind strongly to their natural antigen (K_D_ 7.7 × 10^−9^ M) and show no binding to α-enolase. These results correspond well to those obtained by ELISA (Fig. 1, Table 2).

### 3.3 Identification of the antigenic determinants in α- and β-enolase

Our results show (Fig. 2, Table 2) that anti-α- and anti-β-enolase antibody preparations do not cross-react, therefore must recognize different epitopes. To identify these epitopes and to verify their location on the enolase surface we decided to employ *in silico* approach [45], followed by the synthesis of peptides representing these antigenic determinants and testing their antigenic activity [46].

**Fig. 3:**
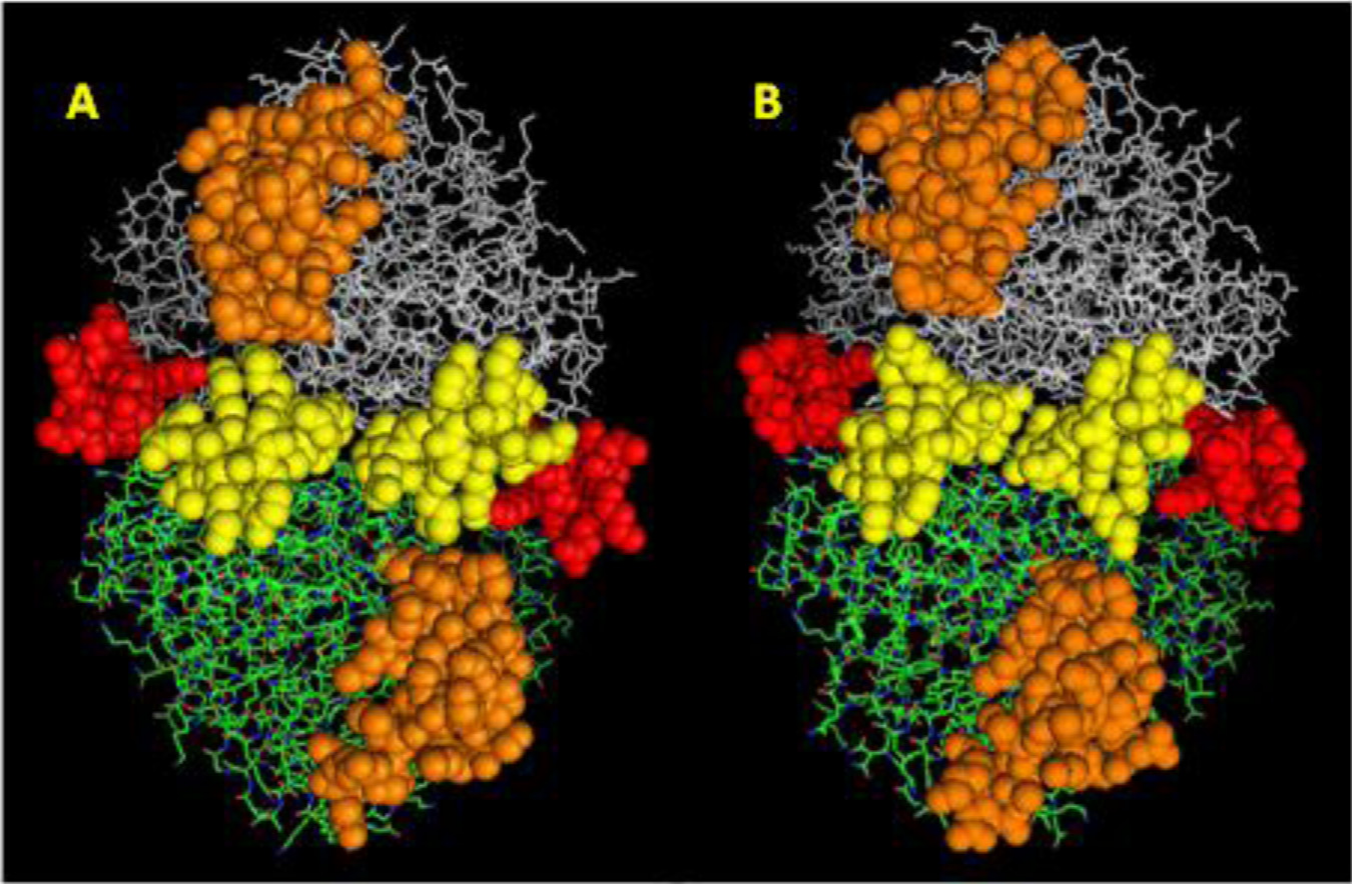
Predicted epitopes on the surface of dimeric human α-enolase (A) and β-enolase (B). The epitopes were labeled as follow: red – epitope 1, yellow – epitope 2, orange – epitope 3. Since both dimers are of the head-to-tail type (shown as white and green monomers), N-terminal epitope 1 appears to be located at the extreme ends of the molecules. This image was created using PyMOL Molecular Graphics System, (Version 1.1) Schrödinger, LLC.

The 3D structures for both α- and β-enolases are well known. The structural data were employed to identify potential conformational epitopes [45] in these two isoenzymes. Three epitopes for both α- and β-enolase were identified and presented in Fig. 3. All three epitopes occupy the same part of the enolase surface and share similar amino acid sequence. The most significant structural differences between two enolases appear in predicted epitopes 1 and 2. The third potential epitope (epitope 3), is larger and somewhat more amorphous than the epitopes 1 and 2. It is rather challenging to test the immunological activity of large surface area such as the one attributed to epitope 3. To simplify this task, we have somewhat arbitrarily divided epitope 3 to N- and C-terminal fragments.

**Fig. 4:**
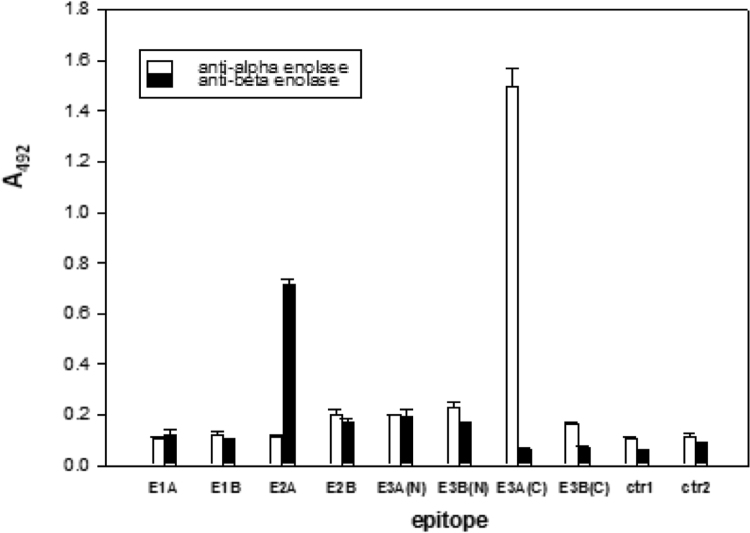
Interaction of polyclonal anti-enolase antibodies with pin-attached peptides representing α- and β-enolase epitopes. Dilution of antibody against β-enolase and α-enolase was 1:100 and 1:1000, respectively. Data are representative of three experiments (n = 3). E1A –α-enolase epitope 1 [D^51^NDKTRYMGK^60^ -GG-*pin*], E1B –β-enolase epitope 1 [D^52^GDKGRYLG K^61^ -GG-*pin*], E2A –α-enolase epitope 2 [N^194^VIKEKYGKDATN^206^ -GG-*pin*], E2B –β-enolase epitope 2 [G^195^VIKAKYGKDATN^207^ -GG-*pin*], E3A(N) –α-enolase epitope 3 (N-terminal fragment) [R^253^SGKYDLDFK^262^ -GG-*pin*], E3B(N) –β-enolase epitope 3 (N-terminal fragment) [R^254^NGKYDLDFK^263^ -GG-*pin*], E3A(C) –α-enolase epitope 3 (C-terminal fragment) [S^263^PDDPSRYISPDQ^275^ -GG-*pin*], E3B(C) –β-enolase epitope 3 (C-terminal fragment) [S^264^PDDPARHITGEK^275^ -GG-*pin*], ctr1 – non-epitope sequence in both α- and β-enolases [K^343^VNQIGSVTES^353^ -GG-*pin*], ctr2 – computer generated random sequence [TQLRAVNHASDF-GG-*pin*].

The peptides with sequences corresponding to predicted epitopes were synthesized on solid phase [46]. Two glycine residues were added as spacers between the C-termini of the peptide and the polyethylene pin. The antibody binding to immobilized peptides was measured by ELISA (Materials and Methods), and the results were shown in Fig. 4. The strongest interaction was observed for the anti-α-enolase interacting with a C-terminal fragment of the α-enolase epitope 3 (S^262^PDDPSRYISPDQ^273^). No interaction was observed between this antibody and the corresponding β-enolase C-terminal fragment of the epitope 3 (S^263^PDDPARHITGEK^274^).

The affinity chromatography purified anti-β-enolase antibody preparation is recognizing epitope 2. Most unexpectedly, however, the anti-β-enolase preparation binds to a peptide bearing sequence corresponding to epitope 2 in α-enolase (N^193^VIKEKYGKDATN^205^-GG-*pin*), while remaining inactive towards peptide derived from β-enolase (G^194^VIKAKYGKDATN^206^-GG-*pin*) epitope 2 (Fig. 4). This finding is very difficult to reconcile with the fact that the same antibody binds to human β-enolase, while remains inactive towards α-enolase (Table 2).

The epitope's 2 Cα chain traces derived from 3D structures of both enolases (α-enolase; 2PSN and β-enolase; 2XSX) are very similar (Supplementary Fig. S4). Most interestingly, N-terminal amino acids in both epitopes form two turns of α-helix. In α-enolase epitope 2 two of these amino acids, N^193^ and E^197^, are known for their propensity to form and to stabilize the secondary structure. On the other hand, N-terminal glycine present in β-enolase's epitope 2 (G^194^VIKAKYGKDATN^206^-GG-*pin*) may have had a destabilizing effect on α-helix in the synthetic peptide [50], while not disturbing the secondary structure in the protein. Unfortunately, replacement of N^193^ with glycine in N^193^VIKEKYGKDATN^205^-GG-*pin* caused only a minor decrease in immunological activity against anti-β-enolase antibodies. At the same time substitution of E^197^ in N^193^VIKEKYGKDATN^205^-GG-*pin* with alanine lead to 70% loss of the immunological activity. Moreover, when A^198^ in β-enolase epitope 2 peptide (G^194^VIKAKYGKDATN^206^-GG-*pin*) was substituted with glutamic acid, 67% increase in activity against anti-β-enolase was observed. Results clearly show that it is a negatively charged glutamic acid that makes this peptide immunologically active. The above-presented results show that bioinformatic identification of the conformational antigenic determinants requires a thorough experimental follow-up. As far as C-fragment of the epitope 3 is concerned, no structural element can be readily identified in either of two enolase isoforms (Supplementary Fig. S5). However, there must be enough structural differences between α- and β-enolase in this region to render selective recognition of α-enolase by anti-α-enolase antibody preparation (Table 2).

The K_D_ value determined by SPR for binding of anti-α-enolase antibodies to E3C-peptide-myoglobin conjugate indicates a highly specific interaction (K_D_ 5.42 × 10^−9^ M), although two orders of the magnitude lower than binding of the same antibody to a native protein. The same conjugate was not recognized by anti-β-enolase antibodies. The anti-β-enolase antibodies, however, interacted with E2A-peptide-myoglobin and showed high-affinity towards this conjugate (K_D_ 1.87 × 10^−8^ M). The results correspond well to those obtained by ELISA for pin-tethered synthetic peptides (Fig. 4).

Further exploration of the precepts governing immunological specificity of the antibodies will be important for developing a selective immunological test for β-enolase.

Our work demonstrates that by using conventional methods, it was possible to obtain highly selective IgG preparations recognizing individual enolase isoforms. Bioinformatic analysis of both α- and β-enolase 3D structures allowed to predict three spatially homologous conformational epitopes, of which two were recognized by affinity-chromatography purified antibodies.

Since the surface determinants only partially explain differences in the antigenic properties of human α- and β-enolase, it might be worth investigating the usefulness of internal antigenic determinants. Such epitopes occupying proteins’ inner structure are important in the mechanism of antigen presentation. In the future, we will expand the use of databases and bioinformatic tools [51] in an effort to explore further immunochemical differences between these two human enolases.
