# Supplementary material for: Experimental and bioinformatic approach to identifying antigenic epitopes in human α- and β-enolases
Source: Biochem Biophys Rep. 2018 Jun 17;15:25–32. doi: 10.1016/j.bbrep.2018.05.008 (PMC6005794; doi:10.1016/j.bbrep.2018.05.008)
Supplement: Supplementary file 2 — Supplementary material [file mmc2.docx]

**Supplementary Data**

**Supplementary Table 1**. Classical purification of α- and β-enolase.

| Fractions | Total protein  [mg] | Total activity  [units] | Specific activity  [units/mg] | Yield  [%] |
| --- | --- | --- | --- | --- |
| Human α-enolase (62 g of kidney) | | | | |
| Crude extract | 7450 | 2652 | 0.35 | 100 |
| (45-67)% (NH_4_)_2_SO_4_  precipitation | 1364 | 2005 | 1.47 | 70 |
| DEAE-Sephadex, pH 9.0 | 46.5 | 804 | 17.3 | 30 |
| CM-Sephadex,  pH 6.0-8.0 | 7.23 | 566 | 78.3 | 21.3 |
| Human β-enolase (110 g of striated muscle) | | | | |
| Crude extract | 5524 | 4218 | 0.75 | 100 |
| Heat treatment, 53-55 °C | 2765 | 3042 | 1.1 | 72 |
| (60-80)% (NH_4_)_2_SO_4_  precipitation | 741 | 2742 | 3.7 | 65 |
| DEAE-Sephadex, pH 9.0 | 118 | 2236 | 18.9 | 53 |
| CM-Sephadex,  pH 6.4-8.5 | 46 | 1434 | 31 | 34 |
| QAQ-Sephadex, pH 9.0 | 8 | 759 | 95 | 18 |

Both α-enolase and β-enolase were purified according to very similar protocols, composed of the following steps: cell extraction, ammonium sulphate precipitation and two consecutive ion-exchange separations on DEAE- and CM-Sephadex. Purification of the human β-enolase, however, required two additional purification steps, i.e., heat treatment of the cell extract (53-55 °C) and an extra ion-exchange chromatography step on QAQ-Sephadex. A detailed purification protocol can be found in Materials and Methods.

**A. B.**


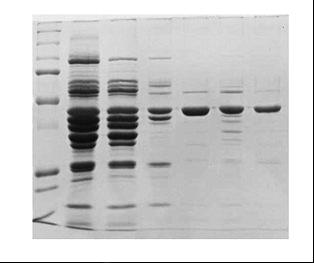


**37**

**150**

**250**

**75**

**25**

**15**

**kDa**

**100**

**50**

**1**

**2**

**3**

**4**

**5**

**6**

**7**


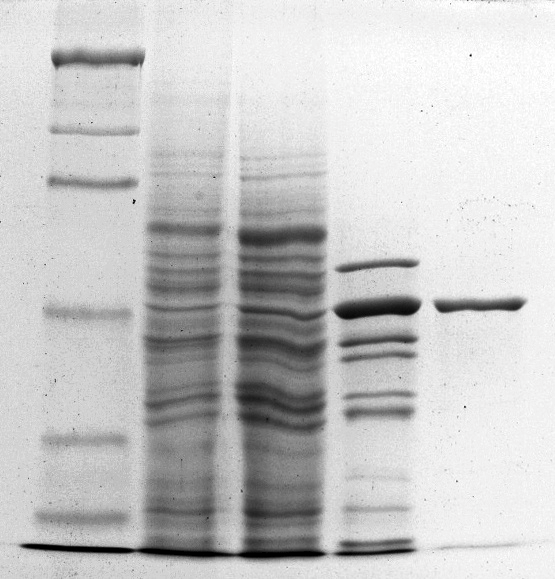


**1**

**2**

**3**

**4**

**5**

**47**

**115**

**204**

**94**

**20**

**10**

**kDa**

**Fig. 1A**

**Supplementary Figure S1.** Purification of α- and β-enolase. Protein pattern after SDS-PAGE (10% gel) of human β-enolase (A) and human α-enolase (B) samples. **Panel A**: lane 1- MW markers, lane 2- crude extract of skeletal muscle (75 μg), lane 3- extract after incubation at 56˚ C (50 μg), lane 4- a sample of 20 μg protein after precipitation with (60-80)% (NH_4_)_2_SO_4_, lane 5- proteins after CM-Sephadex fractionation (15 μg), lane 6- partially purified enzyme after DEAE-Sephadex(20 μg), lane 7- β-enolase after QAE-Sephadex chromatography (4 μg). **Panel B**: lane 1-MW markers, lane 2-proteins after precipitation with (45-67)% (NH_4_)_2_SO_4_ (50 μg), lane 3- kidney crude extract proteins (75 μg), lane 4- proteins after DEAE-Sephadex step (20 μg**)**, lane 5- α-enolase after CM Sephadex (3 μg**)**.


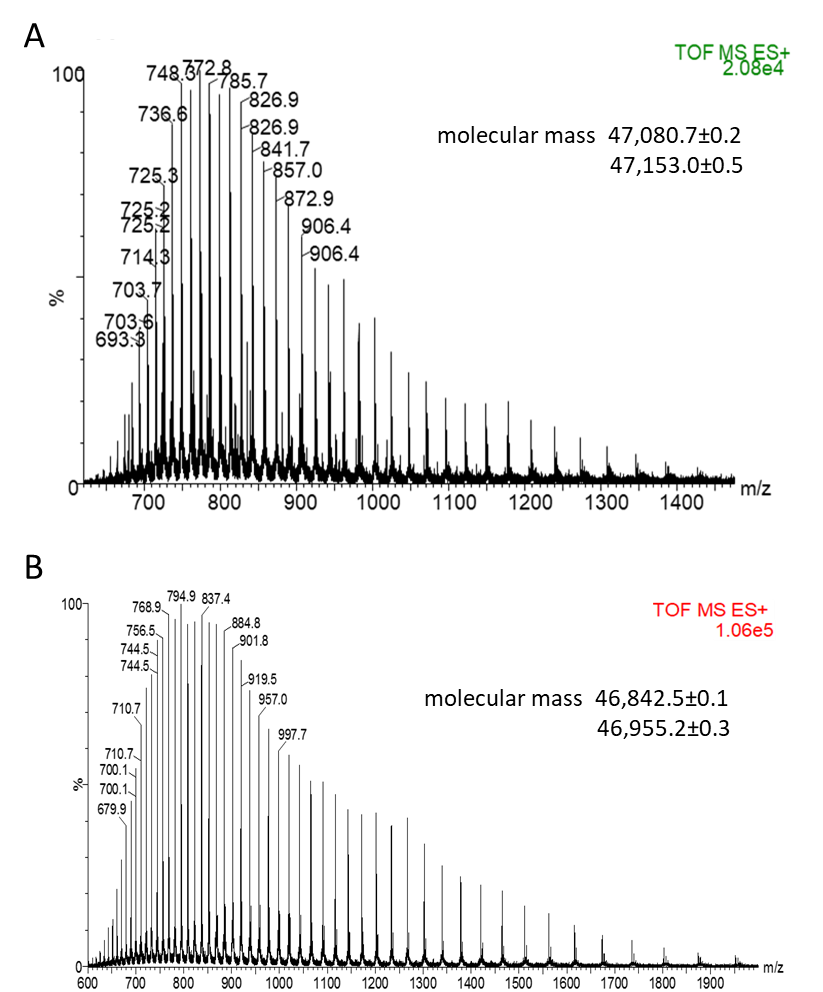


molecular mass 47 080.40±0.2

47 153.00±0.5

molecular mass 46 900.50±0.2

46 845.03±0.3

**Supplementary Figure S2.** Mass spectra of purified human α-enolase (A) and human β-enolase (B).


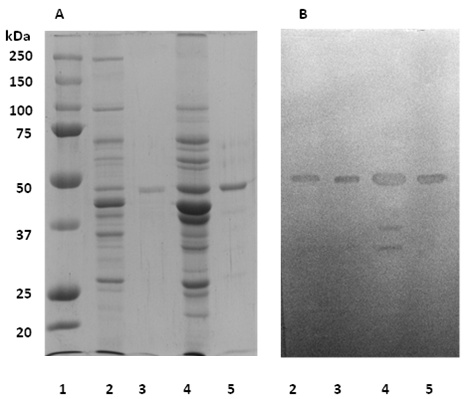


**Supplementary Fig. S3.** Specificity of purified anti-human β-enolase rabbit antibody:

(A) Coomassie-stained SDS-PAGE gel, (B) Western blot developed with anti-human β-enolase rabbit antibody (1:500 dilution). The membranes were treated with the goat anti-rabbit IgG conjugated with alkaline phosphatase secondary antibody (1:5000) and the blot was developed with the alkaline phosphatase substrate Western BlueR (Promega), and the reaction was stopped with water. MW markers (lane 1), crude extracts (20 μg of protein) from human (lane 2) and rat skeletal muscle (lane4), purified human (lane 3) and rat (lane 5) β-enolase (2 µg).


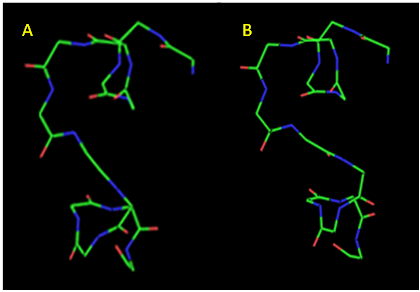


**Supplementary Fig. S4.** Epitope 2 conformation in human α-enolase (A) and β-enolase (B). The Cα chain traces were derived from the X-ray structures of α-enolase (PDB: 2PSN) and β-enolase (PDB: 2XSX) using PyMOL Molecular Graphics System, (Version 1.1) Schrödinger, LLC.


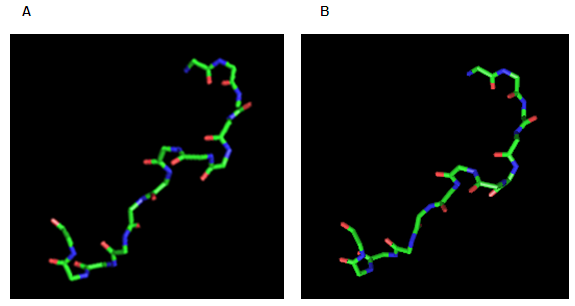


**Supplementary Fig. S5.** Epitope 3 conformation in α- and β-enolase: C-terminal fragment of the epitope 3 in human α-enolase (A) and β-enolase (B). The Cα chain traces were derived from the X-ray structures of α-enolase (PDB: 2PSN) and β-enolase (PDB: 2XSX) using PyMOL Molecular Graphics System, (Version 1.1) Schrödinger, LLC.
